# Crystallization of domains involved in self-assembly of the S-layer protein SbsC

**DOI:** 10.1107/S1744309112042650

**Published:** 2012-11-14

**Authors:** Anđela Đordić, Eva M. Egelseer, Manfred Tesarz, Uwe B. Sleytr, Walter Keller, Tea Pavkov-Keller

**Affiliations:** a Institute of Molecular Biosciences, Karl-Franzens University Graz, Humboldtstrasse 50, 8010 Graz, Austria; b Department of Nanobiotechnology, University of Natural Resources and Life Sciences, Muthgasse 11, 1190 Vienna, Austria; c ACIB (Austrian Centre of Industrial Biotechnology) GmbH, Petersgasse 14, 8010 Graz, Austria

**Keywords:** S-layer, self-assembly, *Geobacillus stearothermophilus*

## Abstract

The Gram-positive bacterium *Geobacillus stearothermophilus* ATCC 12980 is completely covered with a two-dimensional crystalline monolayer composed of the S-layer protein SbsC. In order to complete the structure of the full-length protein, additional soluble constructs containing the crucial domains for self-assembly have been successfully cloned, expressed and purified. Crystals obtained from three different recombinant constructs yielded diffraction to 3.4, 2.8 and 1.5 Å resolution. Native data have been collected.

## Abstract 2

Three different truncation constructs of the S-layer protein SbsC containing domains crucial for self-assembly could be crystallized. Native data were collected for the three crystal forms from crystals that diffracted to 3.4, 2.8 and 1.5 Å resolution.

## 1. Introduction

Crystalline bacterial cell-surface layers, termed S-layers, represent an almost universal feature of archaeal cell envelopes and have been identified in hundreds of different species of bacteria (Sleytr, 1978 ▶; Sleytr & Beveridge, 1999 ▶). They can be regarded as the simplest protein membrane developed during evolution. S-layers, in general, are monomolecular isoporous structures composed of a single protein or glycoprotein species with a molecular mass in the range 40–200 kDa (Sleytr, 1978 ▶; Sleytr & Beveridge, 1999 ▶; Sleytr & Messner, 2009 ▶). Electron-microscopic studies revealed that S-layer lattices can exhibit either oblique (*p*1, *p*2), square (*p*4) or hexagonal (*p*3, *p*6) symmetry, with a centre-to-centre spacing of the morphological units of approximately 3–35 nm (Pavkov-Keller *et al.*, 2011 ▶; Sleytr *et al.*, 1999 ▶).

Despite their ubiquitous appearance and their obvious importance in many prokaryotic organisms, the role of S-layers in nature is not completely clear. However, it is now recognized that S-layer lattices can provide the organism with a selective advantage by fulfilling a broad spectrum of functions (Beveridge, 1994 ▶; Beveridge & Koval, 1993 ▶; Sára & Egelseer, 1996 ▶; Sleytr *et al.*, 1993 ▶, 1999 ▶; Sleytr & Sára, 1997 ▶). Owing to their self-assembly ability, S-layers can be exploited as a patterning element for many nanobiotechnological applications (Egelseer *et al.*, 2010 ▶; Ilk *et al.*, 2011 ▶; Sleytr *et al.*, 2005 ▶, 2007 ▶, 2010 ▶, 2011 ▶).

The protein precursor of the oblique lattice-forming S-layer protein SbsC from *Geobacillus stearothermophilus* ATCC 12980 (Egelseer *et al.*, 1996 ▶) consists of 1099 amino acids including a 30-­amino-acid-long Gram-positive signal peptide (UniProt O68840; Jarosch *et al.*, 2000 ▶). Based on the gene sequence, different N- or C-­terminally truncated S-layer protein constructs have been recombinantly produced and systematically surveyed for their self-assembly and recrystallization properties (Jarosch *et al.*, 2001 ▶). These studies confirmed that the N-terminal part comprising amino acids 31–258 is exclusively responsible for cell-wall binding, whereas the larger, C-­terminal part comprises the self-assembly domain responsible for the formation of the crystalline array. This result has been corroborated by surface plasmon resonance (SPR) and isothermal titration calorimetry (ITC) studies showing that the positively charged N-­terminal region of SbsC binds specifically to a negatively charged secondary cell-wall polymer (SCWP; Ferner-Ortner *et al.*, 2007 ▶; Pavkov *et al.*, 2008 ▶).

The property of S-layer proteins to self-assemble into two-dimensional crystals makes them very demanding candidates for structural studies. The crystallization and/or X-ray structures of several bacterial and archaeal truncated and soluble S-layer constructs that have been reported to date are as follows: N-­terminal and C-terminal parts of SbsC from *G. stearothermophilus* ATTC 12980 (Kroutil *et al.*, 2009 ▶; Pavkov *et al.*, 2003 ▶, 2008 ▶), a truncated derivative of the low-molecular-weight (LMW) S-layer protein from *Clostridium difficile* (Fagan *et al.*, 2009 ▶), the cell-wall-binding domain of the Sap protein from *Bacillus anthracis* comprised of three S-­layer homology (SLH) motifs (Kern *et al.*, 2011 ▶) and polypeptide chain constructs from the archaeal surface-layer proteins from *Staphylo­thermus marinus* (Stetefeld *et al.*, 2000 ▶), *Methanosarcina mazei* (Jing *et al.*, 2002 ▶) and *M. acetivorans* (Arbing *et al.*, 2012 ▶). Recently, the X-­ray structure of the S-layer protein SbsB from *G. stearothermo­philus* PV72/p2 lacking the cell-wall-binding domain was reported (Baranova *et al.*, 2012 ▶). The protein was crystallized in the presence of a nanobody as a crystallization chaperone preventing the self-assembly of the protein into two-dimensional crystals.

**Figure 1:**
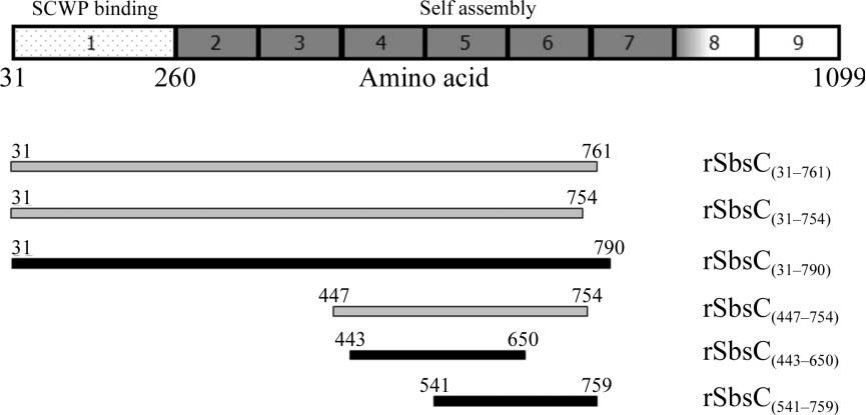
Graphical representation of full-length rSbsC with domains indicated. The SCWP binding domain is marked with dots and domains involved in self-assembly are shown in dark grey. Constructs for which diffraction-quality crystals were obtained are coloured black, whereas constructs that did not yield crystals are coloured light grey.

In order to complete the atomic structure of SbsC, we performed crystallization with different soluble truncation forms containing the domains responsible for self-assembly (Fig. 1 ▶).

## 2. Materials and methods

### 2.1. Cloning, expression and purification

The truncation constructs rSbsC_(31–761)_, rSbsC_(31–754)_, rSbsC_(31–790)_, rSbsC_(447–754)_, rSbsC_(443–650)_ and rSbsC_(541–759)_ were cloned and expressed according to published protocols (Jarosch *et al.*, 2001 ▶; Kroutil *et al.*, 2009 ▶). In brief, the gene sequences encoding the truncations were PCR-amplified using the respective oligonucleotide primers (given in Table 1 ▶), which introduced the restriction sites *Nco*I (including an ATG start codon) and *Xho*I at the 5′ and 3′ ends, respectively. For cloning, the resulting PCR products were ligated with plasmid pET28a+ (Novagen) and the recombinant plasmids were electroporated into *Escherichia coli* TG1 (Stratagene). For expression, the plasmids were established in *E. coli* One Shot BL21 Star (Invitrogen). Purification of the recombinant proteins was performed by fractionated ammonium sulfate precipitation and size-exclusion chromatography as described by Pavkov *et al.* (2003 ▶), except that the lyophilization steps between the chromatography runs were omitted. Purified constructs were dialyzed against 50 m*M* Tris–HCl pH 7.2 and stored at 277 K. The proteins were highly soluble and no degradation was observed within a period of six months.

### 2.2. Crystallization

Initial crystal screening and optimization trials for all constructs were performed with an Oryx8 robot (Douglas Instruments) using commercially available Index (Hampton Research) and Morpheus (Molecular Dimensions Ltd; Gorrec, 2009 ▶) screens. All screens and optimization setups were performed in Douglas vapour-batch plates (Douglas Instruments), which were covered with 3 ml of an oil mixture consisting of paraffin (Merck) and silicone oil (Sigma–Aldrich) in a 3:1 ratio. Drops of 1 µl were pipetted for both initial screens and optimizations. For initial screening, a 1:1 ratio of protein and commercial screening solutions was used. Optimization experiments included variation of this ratio as well as variation of the concentrations of the different screening-solution components. Crystallization plates were incubated at 293 K, with the exceptions of those for rSbsC_(443–650)_ and rSbsC_(541–759)_, which gave better crystals at 289 K.

**Figure 2:**
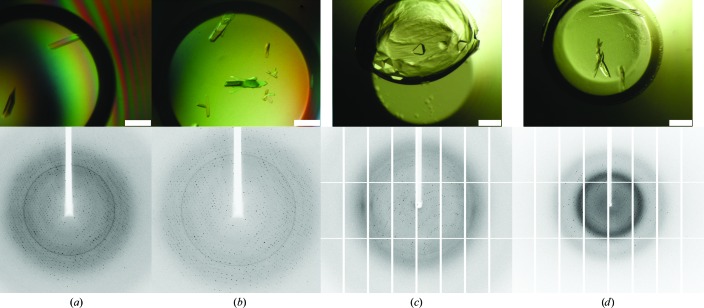
Crystals (upper row) and diffraction images (lower row) of rSbsC_(31–790)_ (*a*), rSbsC_(443–650)_ (*b*), rSbsC_(541–759)_ (*c*) and rSbsC_(541–759)_ in the presence of Ca^2+^ (*d*). The size bars represent 0.2 mm.

Crystallization of rSbsC_(31–790)_ using a protein stock solution at 2.5 mg ml^−1^ in 50 m*M* Tris–HCl pH 7.2 gave several crystals of similar morphology (Fig. 2 ▶ *a*), all of which yielded the same unit-cell parameters after indexing. The best diffracting crystal appeared after two-and-a-half weeks in optimized Morpheus condition 1-46 [5.2%(*w*/*v*) PEG 8000, 10.5%(*v*/*v*) ethylene glycol, 0.05 *M* Bicine/Trizma base pH 8.5 and 0.01 *M* each of 1,6-hexanediol, 1-­butanol, (*RS*)-1,2-propanediol, 2-propanol, 1,4-butanediol and 1,3-­propanediol] with a protein end concentration of 0.75 mg ml^−1^ corresponding to 30% protein solution in the drop.

Crystals of rSbsC_(443–650)_ obtained using a protein stock solution at 5.5 mg ml^−1^ in 50 m*M* Tris–HCl pH 7.2 appeared after five-and-a-half weeks under optimized Index condition No. 40 [0.01 *M* citric acid pH 3.5, 3.0%(*w*/*v*) PEG 3350] with an end protein concentration of 2.25 mg ml^−1^ in the drop (Fig. 2 ▶ *b*).

Several conditions yielded crystals of rSbsC_(541–759)_ with octahedral morphology and with varying size and quality (Fig. 2 ▶ *c*). The protein concentration of the stock solution was 6.4 mg ml^−1^ in 50 m*M* Tris–HCl pH 7.2. Crystals appeared after two-and-a-half weeks in optimized Index condition No. 95 [0.04 *M* potassium thiocyanate, 12.7%(*w*/*v*) PEG MME 2000] with a protein concentration of 3.2 mg ml^−1^ in the drop. Initial optimization setups were repeated with the same protein solution containing 2 m*M* CaCl_2_. Most of these crystals appeared after four weeks. Compared with previous crystals of this truncation form, the new crystals exhibited a different morphology (Fig. 2 ▶ *d*). The plate-like crystals showed mostly twinned or smeared spots in one direction. A complete data set was collected from a thicker plate-like crystal showing isotropic diffraction to 1.5 Å resolution. This crystal grew from a condition consisting of 0.05 *M* potassium thiocyanate, 13.8%(*w*/*v*) PEG MME 2000.

### 2.3. Data collection and processing

Data collection from all crystals was performed at 100 K without additional cryoprotectant, since no ice rings were observed. Data sets from all truncation forms were collected on synchrotron beamlines (EMBL Outstation, Hamburg, Germany and Swiss Light Source, Villigen, Switzerland). Data sets were processed and scaled using the *XDS* program package (Kabsch, 2010 ▶). The unit-cell parameters, assigned space groups and data statistics of the best data set for each truncation form are shown in Table 2 ▶. The number of molecules in the asymmetric unit was derived from the self-rotation function calculated with *MOLREP* (Vagin & Teplyakov, 2010 ▶) as well as the approximate solvent content (Table 2 ▶).

## 3. Results and discussion

The structures of two SbsC C-terminal truncation constructs and the successful crystallization of the N-terminally truncated construct rSbsC_(755–1099)_ have been reported previously (Pavkov *et al.*, 2008 ▶; Kroutil *et al.*, 2009 ▶). The structure of rSbsC_(31–443)_, containing the first three N-terminal domains, has been determined to 2.4 Å resolution (PDB entry 2ra1; Pavkov *et al.*, 2008 ▶). The structure of a longer construct rSbsC_(31–844)_ was at a low resolution and was only partially interpretable. Thus, domain-level information could be derived, but large parts of the structure could only be built as a poly-Ala model. Therefore, two new C-terminal truncation forms of similar length, rSbsC_(31–761)_ and rSbsC_(31–754)_, including domains 1–6, were produced. However, these forms yielded no crystals. Owing to the fact that the partial structure of rSbsC_(31–844)_ showed that the observed ring-like structure is stabilized by extra residues from domain 7 (Pavkov *et al.*, 2008 ▶), a new longer construct rSbsC_(31–790)_ was produced, for which crystals could be obtained. At the same time, constructs consisting of domains 4–5, 5–6 and 4–6 were subjected to crystallization. Diffraction-quality crystals were obtained for the first two constructs, rSbsC_(443–650)_ and rSbsC_(541–759)_. No crystals were obtained for the construct rSbsC_(447–754)_. It appears that the existence of two flexible linkers between the three domains is detrimental to the formation of an ordered crystal lattice.

A search for structures with significant sequence homology to domains 4, 5 and 6 failed. Therefore, molecular replacement was performed using poly-Ala models of individual domains 4, 5 and 6 from rSbsC_(31–844)_ (Pavkov *et al.*, 2008 ▶). For all three truncation constructs, molecular replacement was performed with *Phaser* (McCoy *et al.*, 2007 ▶). Manual inspection of the results obtained using *Phaser* confirmed that rSbsC_(31–790)_ contains one molecule in the asymmetric unit, rSbsC_(443–650)_ contains two molecules in the asymmetric unit and rSbsC_(541–759)_ plus Ca^2+^ contains one molecule in the asymmetric unit. No clear solution could be obtained using data from rSbsC_(541–759)_ without Ca^2+^. We believe that the high flexibility of some loop and/or linker regions in the absence of Ca^2+^ hampers the formation of stable crystal contacts.

Rebuilding and refinement of the structures is in progress and upon completion will yield the complete structure of the full-length SbsC protein.

## Figures and Tables

**Table 1:** Oligonucleotide primer pairs used for PCR amplification of the gene sequences encoding the various rSbsC truncations Overhangs are underlined, restriction sites are shown in bold and start and stop codons are shown in italics.

rSbsC truncation	Primer names	Primer sequence (5′ to 3′)
rSbsC_(31–761)_	rSbsC-31-forw	CGGAATT **CC*ATG*G**CAACGGACGTGGCGAC
	rSbsC-761-rev	GACCG **CTCGAG** *TTA*TTTTAATGTAGTATCGATGACTTCAAC
rSbsC_(31–754)_	rSbsC-31-forw	CGGAATT **CC*ATG*G**CAACGGACGTGGCGAC
	rSbsC-754-rev	GACCG **CTCGAG** *TTA*TTCAACATCTACAGTACCTAGAG
rSbsC_(31–790)_	rSbsC-31-forw	CGGAATT **CC*ATG*G**CAACGGACGTGGCGAC
	rSbsC-790-rev	GACCG **CTCGAG** *TTA*TAAGTTAGCTAGTAACTTAGCTAAAG
rSbsC_(447–754)_	rSbsC-447-forw	CGGAATT **CC*ATG*G**CAGAAGTTAGTGAATTAAAATTAACT
	rSbsC-754-rev	GACCG **CTCGAG** *TTA*TTCAACATCTACAGTACCTAGAG
rSbsC_(443–650)_	rSbsC-443-forw	CGGAATT **CC*ATG*G**ATGAAAAAGCTGCAGAAGTTAG
	rSbsC-650-rev	GACCG **CTCGAG** *TTA*TACTGGTCCGCCAGCAAC
rSbsC_(541–759)_	rSbsC-541-forw	CGGAATT **CC*ATG*G**TTACTAAGACAATCCCTGTGAC
	rSbsC-759-rev	GACCG **CTCGAG** *TTA*TGTAGTATCGATGACTTCAACATC

**Table 2:** Data-collection and processing statistics Values in parentheses are for the outer resolution shell.

	rSbsC_(31–790)_	rSbsC_(443–650)_	rSbsC_(541–759)_	rSbsC_(541–759)_ + Ca^2+^
Beamline	PXI [microfocus], SLS	PXI [microfocus], SLS	PXIII, SLS	PXIII, SLS
Wavelength (Å)	1.0	1.0	1.0	1.0
Detector	MAR CCD 225 mm	MAR CCD 225 mm	PILATUS 2M	PILATUS 2M
Unit-cell parameters (Å, °)	*a* = 57.2, *b* = 98.3, *c* = 109.3, α = γ = 90, β = 94.5	*a* = *b* = 110.3, *c* = 87.2, α = β =γ = 90	*a* = *b* = 106.9, *c* = 110.5, α = β = γ = 90	*a* = 49.1, *b* = 43.8, *c* = 49.7, α = γ = 90, β = 103.8
Space group	*P*2_1_	*P*4_1_2_1_2	*P*4_1_2_1_2	*P*2_1_
Resolution limits (Å)	19.9–3.4 (3.49–3.40)	30–2.6 (2.70–2.60)	49.1–3.4 (3.61–3.40)	48.3–1.54 (1.64–1.54)
*R*_meas_ (%)	11.6 (46.9)	16.4 (44.2)	4.4 (30.9)	5.0 (35.0)
*R*_merge_ (%)	10.6 (42.9)	15.3 (39.2)	3.9 (27.5)	4.2 (29.0)
Total No. of observations	103011 (7638)	94566 (5440)	78216 (12410)	197022 (28684)
Total No. of unique reflections	16664 (1225)	14641 (1365)	16772 (2665)	58519 (9177)
〈*I*/σ(*I*)〉	14.8 (5.1)	10.8 (3.2)	25.7 (5.8)	15.7 (3.0)
Completeness (%)	99.4 (99.8)	85.5 (77.1)	99.4 (97.7)	98.8 (95.7)
Multiplicity	6.2 (6.2)	6.5 (4.0)	4.7 (4.7)	3.4 (3.1)
Wilson *B* factor (Å^2^)	58.6	38.6	96.1	27.2
Matthews coefficient (Å^3^ Da^−1^)	3.8	3.1	3.2	2.3
Molecules per asymmetric unit	1	2	2	1
Solvent content (%)	67.5	60.8	61.7	46.6
